# Tiger stripe: an innocuous Doppler artifact

**DOI:** 10.1186/s44156-026-00104-8

**Published:** 2026-01-21

**Authors:** Biswaranjan Mishra

**Affiliations:** Max Diagnostic, Cuttack, India

**Keywords:** Tiger stripe, Oscillating mass, Red blood cell (RBC) rouleaux, Doppler artifact

## Abstract

**Background:**

Tiger stripes, parallel bands within regurgitant Doppler spectra, are often ascribed to oscillating intracardiac structures. Their frequency and clinical relevance are unclear.

**Methods:**

We retrospectively reviewed 4,567 transthoracic echocardiograms (TTEs) in adults between 18 and 65 years of age for tiger stripes on pulsed-wave (PW)/continuous-wave (CW) Doppler and correlated with valve pathology, presence of oscillating masses, and clinical findings.

**Results:**

Tiger stripes occurred in 5.4% of studies (95% CI 4.8–6.1%), predominantly with mitral and aortic regurgitation jets (MR/AR) and high-velocity tricuspid regurgitation (TR) jets. Stripes were not seen when oscillating masses were present, and were more conspicuous in mild–moderate jets and higher-velocity Doppler spectra. Audible Doppler cooing sound was common; no association with clinical musical murmurs.

**Conclusions:**

Tiger stripes may represent a benign spectral Doppler artifact. In isolation, they should not prompt additional imaging for oscillating masses or be used to grade regurgitation severity.

**Supplementary Information:**

The online version contains supplementary material available at 10.1186/s44156-026-00104-8.

## Introduction

Doppler echocardiography works on the Doppler Effect [1], which means that when a sound source moves toward or away from a receiver, the frequency of the sound heard changes. If the source moves away, the frequency decreases; if it moves toward the receiver, the frequency increases.

In cardiac Doppler imaging, the ultrasound transducer sends sound waves that are reflected by moving red blood cells (RBCs). The transducer receives these reflected signals and uses Fourier analysis [2] to convert them into a spectral Doppler display. This display shows the direction, speed, and amount of blood flow. The vertical axis represents blood flow velocity, the horizontal axis represents time, and the brightness of the signal reflects backscatter intensity, which is related to the relative number of RBCs moving at a given velocity [3]. Signals moving toward the transducer appear above the baseline, while those moving away appear below it. By comparing transmitted and received frequencies, blood flow velocity can be calculated [3].

Sometimes, the Doppler spectrum shows bright, regularly spaced bands above and below the baseline. These are called “tiger stripes” or “zebra stripes.” They are often seen when oscillating structures beneath heart valves move within a regurgitant jet, both in native and prosthetic valve disease [4, 5]. Tiger stripes have also been reported in conditions like Lambl’s excrescences, where similar oscillations are suspected [6]. These stripes are usually accompanied by a high-pitched musical sound on Doppler audio, leading some authors to relate them to musical murmurs caused by vibrating intracardiac structures. However, direct correlation between musical murmurs and tiger stripes has not been established [7], and tiger stripes may also appear without visible oscillating structures [8]. Because prior reports have linked tiger stripes to prosthetic valve malfunction, Lambl’s excrescences, and other oscillating intracardiac structures, their presence has often been interpreted as a marker of potentially serious pathology warranting urgent evaluation [4–7].

Despite these observations, the exact cause, significance, and mechanism of tiger stripes remain poorly understood. Although case reports and small series have described tiger stripes in selected populations, the exact cause, significance, and mechanisms of tiger stripes, particularly their frequency and relevance in an unselected routine echocardiography population, remain incompletely understood. Therefore, this study aims to determine their frequency, clinical importance, and possible mechanism in routine echocardiography practice.

## Method

### Study design

Retrospective, single-center review of consecutive transthoracic echocardiography (TTE) studies performed between 23 January 2024 and 24 January 2025 on a GE Vivid 8 platform were analysed.

### Inclusion and exclusion criteria

Inclusion: Adult patients between 18 and 65 years were eligible. Recorded images were analyzed for presence of tiger stripes in both PW and CW Doppler spectra of valvular flows. Doppler spectra (both PW and CW) were analyzed irrespective of presence or absence of abnormal flows such as stenotic and regurgitant jets.

Exclusion: inadequate Doppler quality precluding spectral interpretation; congenital lesions requiring non-standard windows; intracardiac devices causing uninterpretable artifacts were excluded from the study.

### Doppler settings

TTEs were performed with adult transducer of 1.3 to 4.0 MHz frequency range; sweep speed between 100 and 150 mm/sec; wall filter low; baseline optimized to avoid aliasing; gain adjusted to just fill the spectral envelope; PW sample volume 2–4 mm at the vena contracta/jet origin; CW along jet axis; velocity scale recorded on every image to obtain a complete spectrum.

### Definition of the “tiger stripe” artifact

Tiger stripe was defined as presence of ≥ 2 parallel, echo-dense bands superimposed on a regurgitant Doppler spectrum either PW or CW, at approximately equal spacing, on one or both sides of the baseline, visible in at least two consecutive cardiac cycles.

Images with tiger stripes were further analyzed for etiology and mechanism of valvular regurgitation, presence of oscillating masses such as vegetations and/or flail leaflets in the regurgitant pathways in their TTE images. Doppler recordings of cases with vegetation, chordal rupture and prosthetic valve dehiscence were also examined for presence of tiger stripe in the respective valvular regurgitation spectra.

Clinical data were analyzed for presence of musical murmur in those having tiger stripe. Results were calculated as a percentage of the number of total cases examined and they were additionally sub-categorized according to sex category and etiology of valvular regurgitation expressed as a percentage of total cases.

### Blinded review and statistics

Primary reviewer analyzed all studies blinded to clinical data beyond valve label. The primary reviewer was Dr. Biswaranjan Mishra, who holds a post-doctoral degree in Cardiology from an Indian University having more than 25 years of experience in non-invasive cardiology and echocardiography.

Proportions with 95% CIs was calculated by Wilson method. A limited interobserver validation study was conducted with two qualified cardiologists, blinded to clinical data and each other’s assessments, who independently reviewed a subset of *N* = 100 transthoracic echocardiograms (50 with tiger stripes and 50 without stripe). Readers recorded the presence/absence of tiger stripes. Agreement was quantified using percent agreement and Cohen’s κ (95% CIs).

### Observation

Tiger stripes were noted in 5.4% (*n* = 248) of cases out of 4,567 cases subjected for echocardiography with 95% CI 4.8–6.1% (Wilson method).

**Fig. 1:**
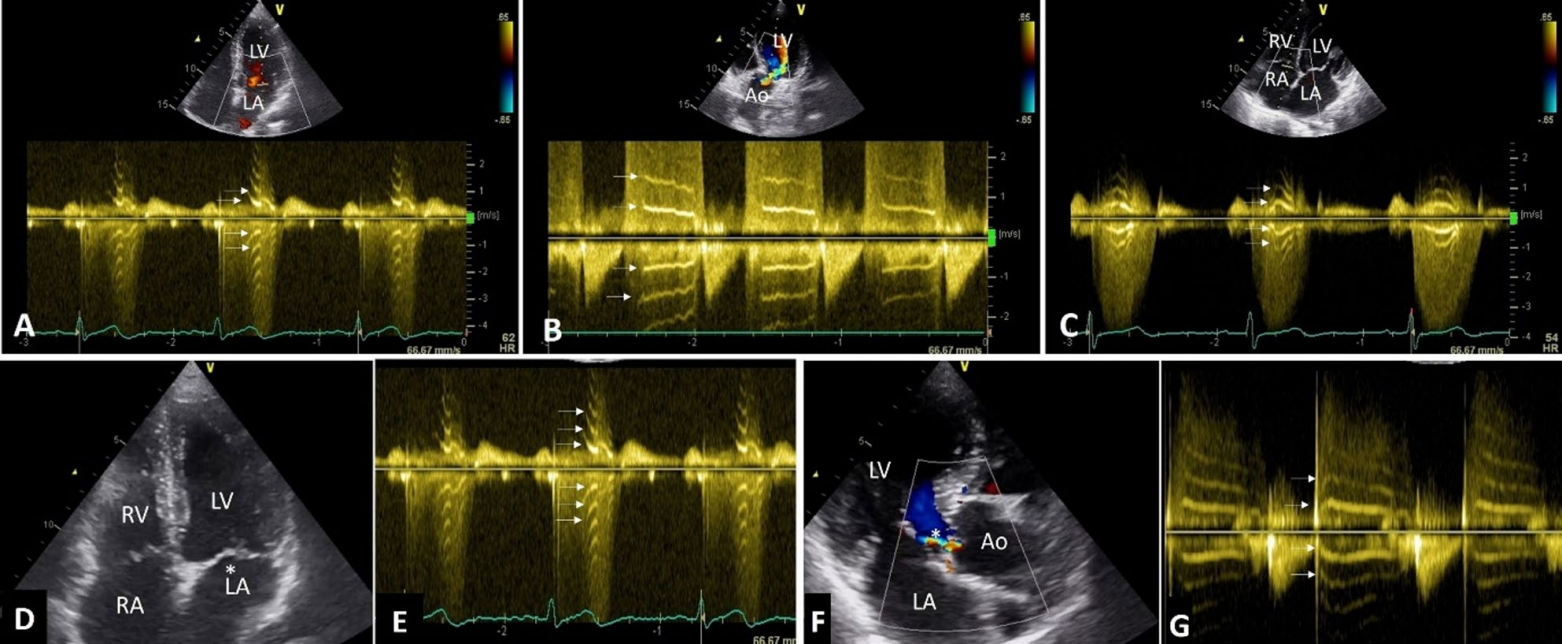
Transthoracic echo showing tiger stripes (horizontal arrows) in left sided regurgitant lesions in (**A**) apical 4 chamber view showing a CW spectrum of mitral regurgitation, (**B**) Apical 5 chamber view showing CW Doppler spectrum of aortic regurgitation. (**C**) Apical 4 chamber showing high velocity tricuspid regurgitant jet due to pulmonary hypertension. (**D**-**G**). Tiger stripes without oscillating structure in regurgitant pathway. (**D**) Apical 4 chamber view does not show any oscillating mass (*) in a case of mild mitral regurgitation (see video 1 and video 2). (**E**) CW Doppler spectrum of the same case shows tiger stripes (arrows) (**F**) No oscillating mass is seen in aortic regurgitation pathway (*), (**G**) Same case showing tiger stripes in mild aortic regurgitation spectrum (arrows), (see video 3). RV-Right ventricle, LV-Left ventricle, RA-Right atrium, LA-Left atrium, Ao-Aorta

Tiger stripes occurred in left side of heart more often such as mitral regurgitation (MR) and aortic regurgitation (AR) where the regurgitant spectral velocity is high (Fig. 1A, B). Stripes were seen in 75.4% (*n* = 187) of MR, 17.7% (*n* = 44) of AR and in 6.8% (*n* = 17) of cases with hypertensive tricuspid regurgitation (TR) secondary to pulmonary hypertension (Fig. 1C). Stripes were not detected in non-hypertensive TR, pulmonary regurgitation, stenotic lesions and diastolic flow across atrioventricular valves. Number of cases of tiger stripes having MR, AR, TR with their sex distribution and mechanism of regurgitation are enumerated in Table 1.

**Table 1:** Showing total number of consecutive patients examined, number having tiger stripe and the types of different regurgitant lesions and their mechanisms

Number of patients examined	Tiger stripe present	Mitral Regurgitation	Aortic Regurgitation	Tricuspid Regurgitation
4,567	*n* = 248 (5.3%)	*n* = 187 (75.4%)	*n* = 44 (17.7%)	*n* = 17 (6.8%)
Male	*n* = 119 (48%)	*n* = 77 (41.2%)	*n* = 15 (35%)	*n* = 07 (41%)
Female	*n* = 129 (52%)	*n* = 110 (58.8%)	*n* = 29 (65%)	*n* = 10 (59%)
Etiology		MVP *n* = 53 (28%)	Sclerotic AV *n* = 28 (63.6%)	Group 2 PH *n* = 11 (64.7%)
		RHD *n* = 45 (24%)	BAV *n* = 6 (13.6%)	COPD *n* = 5 (29.4%)
		Secondary *n* = 89 (47.6%)	RHD *n* = 10 (22.7%)	PAH *n* = 1 (5.8%)

RHD-Rhematic heart disease, MVP-Mitral valve prolapse, DCM-Dilated cardiomyopathy, CAD-Coronary artery disease, AV-Aortic valve, BAV-Bicuspid aortic valve, PH-Pulmonary hypertension, group 2 PH-Pulmonary hypertension due to left heart disease, COPD-Chronic obstructive pulmonary disease, PAH-Pulmonary arterial hypertension

**Fig. 2:**
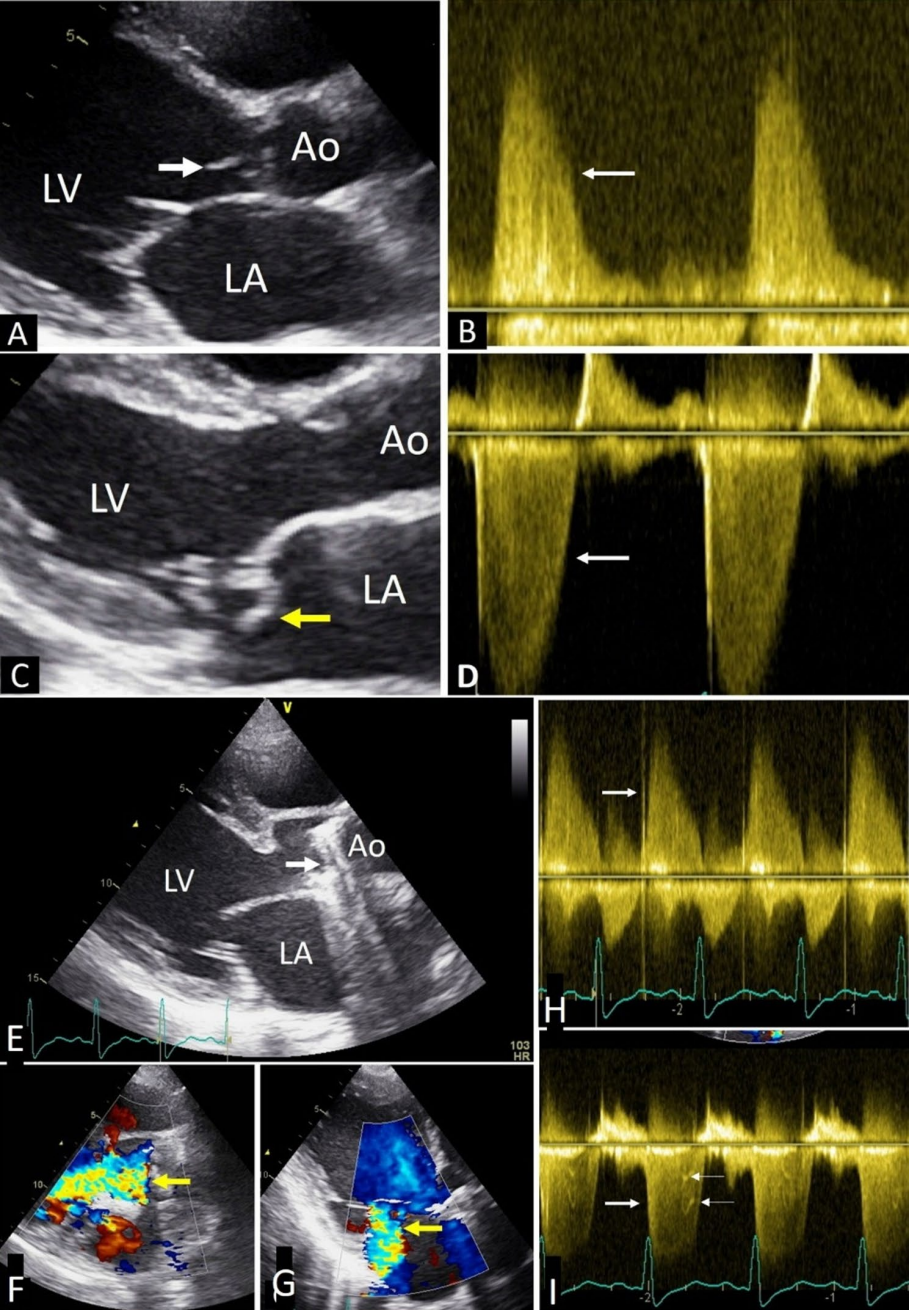
Tiger stripes are absent in cases with oscillating structures in flow pathways. (**A**) Vegetation (white arrow) in aortic valve seen in parasternal long axis view of trans thoracic echo (see video 4). (**B**) CW Doppler spectrum of acute aortic regurgitation in same patient in apical 5 chamber view (arrow) without any stripe. (**C**) Ruptured chordae prolapsing to left atrium in systole (yellow arrow) seen in parasternal long axis view (see video 5), (**D**) CW Doppler spectrum of the same patient in apical 4 chamber view have no stripe seen (arrow). (**E**-**I**). A case of aortic mechanical prosthetic valve dehiscence, (**E**), arrow shows the prosthetic valve in a 2-dimensional transthoracic echocardiographic image in parasternal long axis view, (**F**) and (**G**), arrows show the aortic regurgitant and mitral regurgitant jets respectively in long axis view. (**H**), CW Doppler spectrum of acute aortic regurgitation spectrum does not show tiger stripe (arrow). (**I**), tiger stripes are present in the CW Doppler spectrum of mitral regurgitant jet (small arrows). LV-Left ventricle, LA-Left atrium, Ao-Aorta

None of the cases with tiger stripes were associated with any kind of oscillating structure in the regurgitation pathway. At the same time tiger stripes were not noted where oscillating structures were present in regurgitant flow pathway such as presence of vegetation or ruptured chordae with flail mitral leaflets. Oscillating structure such as vegetations were noted in 3 cases of AR and 5 cases of MR in native valves. Figure 1D-G, Video 1, Video 2 and video 3 show patients with mild MR and mild AR without any oscillating mass in the regurgitant flow pathways but presence of tiger stripes in the Doppler spectra. Video 4 show vegetation in aortic valve and video 5 show ruptured chordae tendinea in flow pathways of AR and MR respectively but Doppler recordings of the same did not show tiger stripes (Fig. 2). In a case of aortic mechanical prosthetic valve dehiscence, the stripes are absent in the AR spectrum whereas stripes are seen in the same case with the MR spectrum (Fig. 2, E-I).

**Fig. 3:**
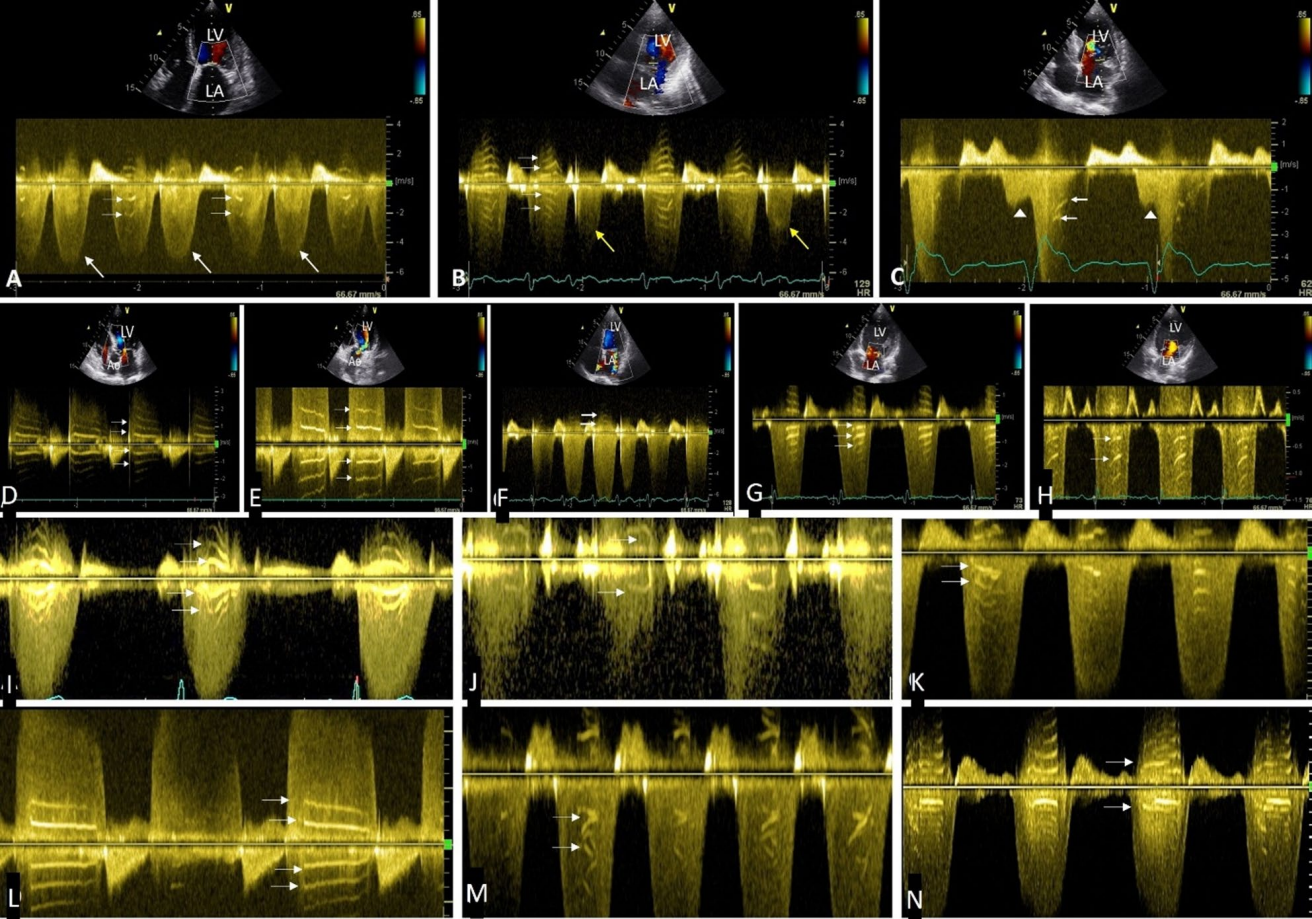
Doppler spectra showing tiger stripes in different situations. (**A**), stripes are seen in sinus beats (horizontal arrows) but absent in premature beats (white arrows) in a patient with bigeminal rhythm and mitral regurgitation (MR), (**B**), MR spectrum in smaller cycles during atrial fibrillation (yellow arrows) do not show stripes but stripes (horizontal arrows) are seen in longer cycles. (**C**). CW Doppler in diastolic MR (arrow heads) in a patent with first degree heart block do not have stripes, whereas stripes (horizontal arrows) are present in systolic MR spectrum. (**D**). Tiger stripes stand out prominently in a mild aortic regurgitation CW Doppler (arrows) with a relative faint spectrum. (**E**). Stripes are seen on either side of baseline like mirror image in a PW Doppler spectrum of aortic regurgitation obtained from apical 5 chamber view with sample volume just below the aortic valve. (**F**). In a relatively denser spectrum the stripes are only seen on opposite side of baseline (arrow) in CW Doppler spectrum of mitral regurgitation obtained from apical 4 chamber view. (**G**). Stripes in CW Doppler in a faint mitral regurgitation spectrum (Arrows), (**H**). PW Doppler in the same patient also shows the stripes (arrows). I-N, tiger stripes occurring in different shapes (arrows) in regurgitant Doppler spectra, in curved lines (**I**), short lines (**J**, **K**), horizontal lines (**L**), oblique lines (**M**), overlapping lines (**N**). LV-Left ventricle, LA-Left atrium, Ao-Aorta

Stripes were not seen in cycles having lower velocity such as premature beats, smaller cycles during atrial fibrillation and during diastolic MR or TR where regurgitant velocity is low, even in presence of such stripes in cycles where velocity of regurgitant flow is high in the same patient (Fig. 3A-C). Stripes were more prominent when the regurgitation is mild producing a relatively faint spectrum over which dense band like shadows stand out (Fig. 3D). These were seen on either side of baseline in a mirror image pattern (Fig. 3E). In severe regurgitation with denser spectrum stripes were seen on the opposite side of the baseline (Fig. 3F). It was seen in both PW and CW Doppler (Fig. 3G, H). In addition to parallel horizontal lines, stripes of various shapes were present including curved lines, horizontal lines, short lines, oblique lines and overlapping lines (Fig. 3I-N).

All cases with tiger stripes were associated with a high-pitched cooing or whistling sound of the returning audible signal of Doppler, but not associated with clinical musical murmur on auscultation.

Interobserver validation was done on a limited subset (*n* = 100). Agreement was quantified using percent agreement and Cohen’s κ (95% CIs). Interobserver agreement for tiger stripe detection was observed to be 91% with κ = 0.82 (95% CI, 0.73–0.91), indicating almost perfect agreement.

## Discussion

There are isolated case reports of tiger stripes in regurgitant lesions in both native and prosthetic valves due to oscillating structures in the regurgitant pathways [4]. H. Feigenbaum et al. attributed tiger stripe to a prosthetic valve malfunction and suggested that oscillating structure in flow pathway generates the characteristic stripes and the whistling or cooing returning Doppler signal [5]. G Davogustto et al. attributed tiger stripes to Lambl’s excrescences [6]. Due to such audible signal stripes were correlated with clinical musical murmur. HN Sabbah et al. studied the hemodynamic determinants, frequency and amplitude of a musical murmur produced by a regurgitant mitral bioprosthetic valve, but their study did not correlate tiger stripes in Doppler examination of these patients [7]. Study on frequency, significance and mechanism of such a finding on Doppler echo is not found on a reasonable literature search. To our knowledge, however, systematic data on the frequency, clinical significance and mechanisms of tiger stripes in a broad, consecutive TTE population remain limited, with most prior descriptions arising from selected case reports or small series.

Our observation on tiger stripes reveal that these are not uncommon, present in 5.4% (*n* = 248) of consecutive cases referred for echocardiography over a period of one year. Stripes are seen in left sided regurgitant lesions such as MR, AR and in TR due to pulmonary hypertension where the velocity of regurgitant flow is relatively high. Stripes are seen to be absent in low velocity flows in premature cycles, in atrial fibrillation and in diastolic MR, where the stripes are seen in jets having higher velocities in the same Doppler recordings. It is seen both in CW and PW Doppler spectra on both sides of baseline. These are more prominent in mild to moderate regurgitant lesions. In severe lesions denser spectrum obscures the stripes which in such cases are seen opposite to the baseline. Interestingly none of the patient with tiger stripe had oscillating structures in their regurgitant pathway. At the same time, we did not find tiger stripe in patients where oscillating masses were present in the regurgitant pathway such as vegetations, ruptured chordae and prosthetic valve dehiscence, neither tiger stripes were confined to any particular etiology. These were seen in both primary and secondary MR and in AR due to rheumatic, sclerotic and congenital bicuspid aortic valve.

The differences between our findings and prior reports that implicate oscillating structures as the cause of tiger stripes [4–7] may reflect several factors. Earlier descriptions have typically involved highly selected patients with prosthetic valve dysfunction or discrete oscillating lesions, in whom tissue motion may dominate the Doppler backscatter pattern. In contrast, our consecutive cohort likely captures a wider spectrum of regurgitant lesions, in which high-velocity flow and blood element behaviour (rather than tissue oscillation) may be the predominant determinants of the observed spectral pattern. Differences in Doppler settings, imaging platforms, and case mix may also contribute to the apparent discrepancy.

Presence of tiger stripe did not add to estimation of severity neither mechanism of valvular regurgitation. Further investigation to rule out oscillating mass is not warranted basing on stripes alone. Recent guideline on assessment of prosthetic valve dysfunction also did not mention about tiger stripe as an indication of valve dysfunction [9].

### Proposed hypothesis

**Fig. 4:**
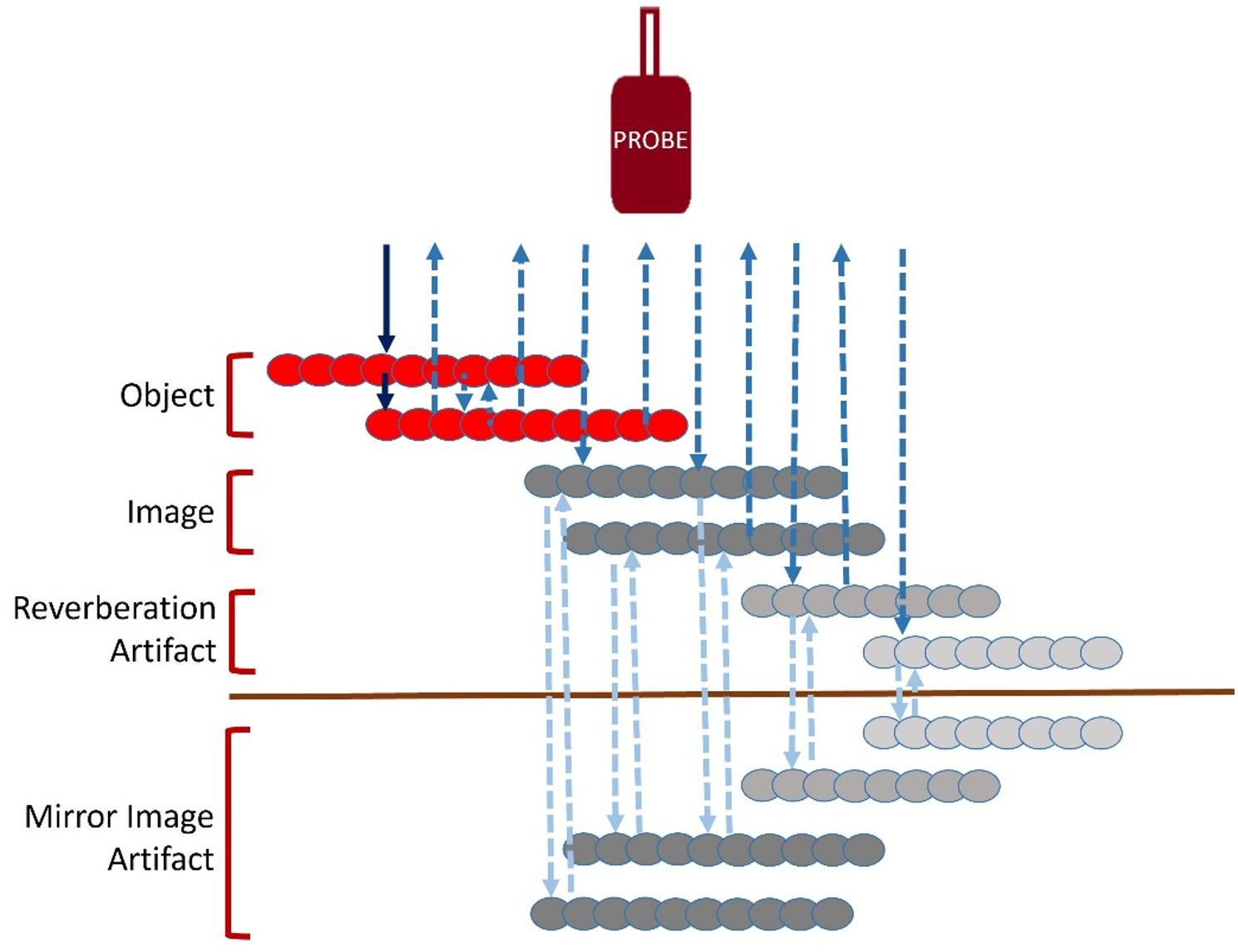
Hypothetical mechanism of tiger stripes. Critically placed rouleax in parallel (object) act as strong reflecting surfaces generating echodense images, parallel objects reflect ultrasound between them creating reverberation artifacts, further reflection from images and artifacts in both direction (dotted arrows) are responsible for causing mirror image artifact

Here we propose a possible explanation for these stripes. During valvular regurgitation the blood flow encounter a relatively small orifice in respect to its flow in the ventricle or great artery. There is a flow convergence proximal to the orifice [10]. Flow convergence means there is transient slowing of blood flow before it enters the regurgitant orifice. It is known that slowing of blood flow result in rouleaux formation by the RBCs [11]. Rouleaux are aggregates of RBCs like stacks of coins. During Doppler examination rouleaux may act differently while reflecting the received ultrasound when compared to individual RBCs in flowing blood. Rouleaux are known to produce echo-dense shadows and are reflector of ultrasound waves [12, 13]. The rouleaux may act a source of reflecting sound causing the dense band like shadow. Strong reflection from parallelly placed rouleaux relative to its surrounding blood may cause reverberation artifacts producing parallel images at equidistant plausibly producing the characteristic stripes [14]. Signal opposite to baseline can be possible due to mirror image artifacts. Doppler angle near 90^o^ to the horizontally placed rouleaux may cause directional ambiguity which may result in spectral display on both sides of baseline [15]. Therefore, tiger stripes may represent a combination of reverberation and mirror image artifacts (Fig. 4) with a cooing or whistling sound heard in the audible Doppler signal is possible due to returning high frequency sound waves making the characteristic high-pitched sound. It is not a consistent finding as it may depend on a relative position of RBC rouleau and Doppler beam, possibly the alignment and timing is critical in the generation of such artifacts. Further as we observed, its appearance in the spectrum is more prominent when the background spectrum is relatively less dense making it commoner in mild to moderate regurgitant lesions. Higher pressure difference across proximal and distal chambers in high velocity regurgitant lesions may be responsible for the sudden deceleration of flow and more pronounced rouleaux formation resulting in tiger stripe artifact.

Therefore, tiger stripes may represent a reproducible Doppler artifact rather than flow modulation by oscillating tissue. Stripe appearance is plausibly due to reverberation and/or mirror-image phenomena in the presence of strong scatterers. Aggregation of red blood cells (rouleaux) during proximal flow convergence could increase backscatter and create parallel bands; however, this mechanism remains hypothetical. This proposed mechanism is exploratory and was not directly tested in the present study, and should therefore be regarded as speculative.

### Clinical implication

Recognizing and understanding artifacts is crucial for accurate diagnosis and to avoid misdiagnosis of serious pathological conditions. Classical descriptions of artifacts such as reverberation, acoustic shadowing, mirror-image, artifacts related to ultrasound beam properties, and those generated by strong intracardiac reflectors such as implanted devices provide alternative, non-pathological explanations [16]. Recognition of tiger stripe as one of the artifacts may help avoid unnecessary investigations for presumed oscillating masses when no corroborating imaging signs exist and moreover, presence of tiger stripes should not be used neither to grade severity or infer mechanism.

### Limitations of the study

Single center, retrospective study with potential selection bias. Single-platform acquisition; limited interobserver sample; general conclusion with speculative mechanism not directly tested. We did not perform a formal systematic or scoping review of the literature; prior reports were identified pragmatically, and some published series on tiger stripes may therefore not have been captured.

## Conclusion

Tiger stripes in Doppler echocardiography are traditionally thought to be result of intracardiac oscillating structures in the regurgitant blood flow pathways therefore representing clinically grave situation mandating further evaluation on a relatively urgent basis and have often been interpreted as potential markers of significant pathology requiring further evaluation [4–7]. But present study showed it is not uncommon in routine echocardiography and is a benign condition representing a kind of spectral Doppler artifact in left sided regurgitant lesions and in TR due to pulmonary hypertension. Stripes alone does not mandate further imaging to look for oscillating mass in regurgitant pathway.

Video 1: 2D Echo in apical 4chamber view of the patient in Fig. 2a with tiger stripe (Fig. 2b) showing normal mitral valve and no oscillating mass in the sub-valvular area. 

Video 2: Color Doppler in apical 4chamber view of the same patient as in video 1 showing mild mitral regurgitation.

Video 3: Color Doppler in apical 3chamber view of the patient with mild aortic regurgitation but no oscillating mass in the sub-valvular area (Fig. 2c) showing tiger stripe (Fig. 2d) in CW Doppler.

Video 4: 2D Echo in parasternal long axis view showing vegetations in aortic valve which present as oscillating mass in aortic regurgitant flow pathway (Fig. 3a) with no tiger stripe on CW Doppler in 5 Chamber view (Fig. 3b).

Video 5: 2D Echo in parasternal long axis view showing prolapse of ruptured chordae to anterior mitral leaflet presenting as oscillating mass in mitral regurgitant flow pathway (Fig. 3c) but no tiger stripe (Fig. 3d).

## Electronic Supplementary Material

Below is the link to the electronic supplementary material.

**Supplementary Material 1:** Video 1

**Supplementary Material 2:** Video 2

**Supplementary Material 3:** Video 3

**Supplementary Material 4:** Video 4

**Supplementary Material 5:** Video 5

## Data Availability

No datasets were generated or analysed during the current study.
